# Internet Use and Self-Rated Health: The Mediating Role of Physical Exercise

**DOI:** 10.3390/healthcare13070714

**Published:** 2025-03-24

**Authors:** Fangmei Zhu, Bowen Tan, Yi Jang

**Affiliations:** 1 School of Public Health, Chongqing Medical University, Chongqing 401331, China; 2022110640@stu.cqmu.edu.cn (F.Z.); tanbowen015@163.com (B.T.); 2 Research Center for Medical and Social Development, Chongqing Medical University, Chongqing 401331, China

**Keywords:** Internet use, self-rated health, physical exercise, mediation effect

## Abstract

**Background:** The rise in digital engagement has positioned self-rated health (SRH) among Chinese adults as a pressing public health challenge. This study employs cross-sectional data to analyze the mechanisms by which Internet use impacts SRH outcomes, with particular emphasis on elucidating physical exercise’s moderating role within this dynamic relationship. **Methods:** The data were obtained from the 2021 China General Social Survey (2021CGSS). The analytical sample comprised 7582 participants. An ordered probit model was used to analyze the relationship between Internet use, physical exercise, and self-rated health. To assess the robustness of the results, an alternative model and a substitute independent variable were employed. The Karlson–Holm–Breen (KHB) method was applied to examine the mediating role of physical exercise. **Results:** Significant correlations were found between Internet use, physical exercise, and self-rated health (*p* < 0.01). Physical exercise partially mediated the positive effect of Internet use on self-rated health, accounting for 9% of the total effect. **Conclusions:** This study suggests a positive relationship between Internet use and self-rated health, with physical exercise playing a mediating role. Expanding Internet access and reducing the digital divide across regions and age groups may contribute to better public health outcomes.

## 1. Introduction

The rapid evolution of Internet technology has driven growth in global Internet users. As of December 2023, China reported 1.092 billion Internet users, reflecting a national penetration rate of 77.5% [1]. This digital expansion profoundly influences socioeconomic dynamics, particularly health-related behaviors, by facilitating access to online health services such as medical consultations, appointment scheduling, and medication procurement [2,3]. The Chinese government has attempted to use Internet-based strategies to enhance public health outcomes. The “Healthy China” strategy is a key developmental goal of China’s 14th Five-Year Plan and the Vision 2035. In the digital age, digital literacy is a fundamental indicator of national quality and plays a crucial role in public health [4]. Exploring the theoretical mechanisms and impacts of digital literacy on individual health is of great practical significance, as it contributes to advancing both the “Digital China” and “Healthy China” initiatives [5,6].

Extensive research demonstrates that Internet engagement positively influences both physical and psychological well-being [7,8]. Enhanced social participation through digital platforms strengthens interpersonal relationships, mitigates loneliness, and reduces risks of depression, anxiety, and cognitive decline [9,10]. Furthermore, online interactions foster perceived health improvements by providing social support [11,12]. As a primary conduit for health information dissemination, the Internet bridges resource gaps, enabling equitable access to medical knowledge and services [13,14]. However, Internet use is also associated with a number of side effects. Internet use can lead to increased social comparison, heightened feelings of relative deprivation, and subsequent anxiety and depression [15,16]. Internet behavior compresses direct communication between groups, reduces emotional expression, and affects the preservation of interpersonal relationships [17,18].

Regarding physical exercise, the World Health Organization (WHO) recommends adults engage in 150–300 min of moderate-intensity or 75–150 min of vigorous-intensity physical activity weekly [19]. The Chinese government emphasized the significance of exercise in its “National Fitness Plan (2021–2025)” issued in 2021. How to promote the continued transformation and upgrading of the “Internet + fitness” movement is the focus of attention of China’s public health policy [20]. Physical exercise is an indispensable part of social life, and regular physical activity can improve the quality of life and sense of well-being. Exercise and SRH are correlated in numerous research [21,22,23]. Zhang (2021) [20], using data from the 2017 CGSS, found that physical exercise mediated the association between Internet use and improved mental health outcomes in older adults. In this context, the connection between Internet use and exercise has emerged as an important field of research. Some researchers have found that Internet use significantly increases the frequency of participation in physical exercise [24]. Guo et al. (2022) found that Internet use increases opportunities for acquiring information, promotes community engagement, and enhances knowledge of exercise and sports, thus encouraging physical activity among older adults [24]. However, other academics suggest that using the Internet for gaming can cut into the amount of time spent exercising, thereby suppressing the frequency of exercise [25].

Current academic discourse reflects persistent divergence regarding the health implications of Internet engagement, with conflicting evidence on its potential benefits or risks. While empirical studies confirm physical activity’s capacity to enhance health perceptions, scant research has systematically examined exercise as a mediating mechanism linking digital behaviors to self-rated health outcomes. Addressing this gap, our analysis leverages 2021 CGSS data to investigate how physical exercise modulates the association between Internet use and subjective health evaluations in Chinese adults, extending prior theoretical frameworks through empirical validation.

## 2. Materials and Methods

### 2.1. Data and Sample

The data for this study came from the 2021 China General Social Survey (CGSS), a nationally representative longitudinal social survey initiated by the China Center for Survey and Data of Renmin University of China in 2003. Utilizing a multi-stage stratified sampling framework, the CGSS collects comprehensive socio-demographic, economic, and health-related data across 19 provincial-level administrative regions, encompassing urban and rural populations. The 2021 wave included 8148 valid responses from adults aged 18 years and above. After excluding cases with incomplete data through listwise deletion, the final analytical sample comprised 7582 participants (93.1% retention rate). Given the ordinal nature of both the dependent variable and key independent variables, an ordered probit regression model was selected for analysis. This approach avoids restrictive assumptions of linear regression while providing unbiased estimates for categorical outcomes.

### 2.2. Variables

The dependent variable, self-rated health (SRH) was operationalized using a validated single-item measure: “How do you feel about your current physical health?” [13,26,27]. The responses were scored on a 5-point Likert scale (1 = poorest to 5 = optimal), where higher values denote better-perceived health.

Independent variable. Internet use (INT) was dichotomized based on the question, “How often did you use the Internet in the past year?” (0 = “never”; 1 = other responses). To strengthen validity, a proxy variable, “leisure Internet use” (Leisure) was constructed from the item “Did you frequently use the Internet during leisure time in the past year?” (1 = rare to 5 = daily).

Mediating variable. Physical exercise frequency was assessed via “How often did you engage in leisure-time physical exercise in the past year?” (1 = never to 5 = daily).

Control variables. Previous studies found significant individual differences in self-rated health according to gender, age, marriage, and education [28,29]. Hukou, a classification based on household registration, is a crucial dimension to take into account in the Chinese context since it may have an impact on how well Chinese people perceive their health [30]. Therefore, this study included gender, age, household registration, education level, marital status, employment status, and objective health status in the analysis. To ensure the robustness of the results, provincial dummy variables were also introduced.

## 3. Results

### 3.1. Descriptive Analysis

According to Table 1, most participants (53.49%) said they were in good health. In total, 71.84% of participants used the Internet, and 63.28% of the population used the Internet frequently in their leisure time. In total, 39.87% of the respondents exercised regularly, compared to 60.13% who never exercised or rarely exercised. The respondents were divided into three age groups according to WHO classification: youth (18–44), middle-aged (45–59), and elderly (60 and above). These groups accounted for 33.98%, 30.26%, and 35.76% of the sample, respectively. Men comprised 45% of the sample and women comprised 55% of the sample. In total, 60% of participants were from rural, and 40% were from urban areas. In addition, 72% of those included were married.

### 3.2. Correlation Analysis of Key Variables

The Spearman correlation coefficients between the major variables are displayed in Table 2. Physical exercise, Internet use, and leisure Internet use were positively associated with self-rated health. Internet use was correlated with leisure Internet use. Physical exercise had a positive association with Internet use and leisure Internet use.

### 3.3. Multicollinearity Analysis

A multicollinearity assessment was conducted before the empirical analysis. As shown in Table 3, the variance inflation factors (VIFs) are all below 2, well under the critical value of 10, indicating no multicollinearity in the data.

### 3.4. Analysis of Regression Results

#### 3.4.1. Baseline Regression Results

As shown in Table 4, Model 1 only included Internet use and the dummy variables, and Internet use significantly affected self-rated health (β = 0.656, *p* < 0.001). This relationship remained significant (β = 0.119, *p* < 0.001) after including control variables. From the perspective of individual characteristics, age was negatively associated with self-rated health. Rural residents (β = 0.0659, *p* < 0.05) were healthier compared to urban residents, which is consistent with many previous studies [13,20]. The residents who had work experience showed a decline in their self-rated health compared to those who had never worked, and the unemployed were more likely to have health problems (β = −0.224, *p* < 0.001). There are two possible explanations for this result: either poor health may be the cause of unemployment, or unemployment may lead to a decline in economic level and lower quality of life, which in turn affects an individual’s self-rated health. Objective health was positively associated with self-rated health. The higher the level of education, the better the self-rated health. Gender and marital status had no significant correlation with self-rated health.

#### 3.4.2. Robustness Test

As shown in Table 5, we replaced the original model with the Ologit model to further assess the results for robustness [13], and an alternative independent variable was tested [24]. If the regression results remain consistent and stable, it indicates that the findings of this study are relatively robust. Models 5 and 6 present the Ologit regression results. Internet use had a significant impact on residents’ self-rated health (β = 1.152, *p* < 0.001; β = 0.215, *p* < 0.001), consistent with expectations. Middle-aged and young adults, rural residents, unmarried individuals, those without work experience, and residents with higher education levels tended to report better self-rated health. Models 7 and 8 show the regression results after replacing the variable of Internet use with leisure Internet use. The frequency of leisure Internet use was significantly correlated with self-rated health (β = 0.177, *p* < 0.001; β = 0.0351, *p* < 0.001). These results confirm the robustness of the findings.

#### 3.4.3. Heterogeneity Analysis

Depending on personal characteristics, Internet use has different effects on health [31]. Table 6 reports the impact of Internet use on individuals’ self-rated health by age and hukou. Internet use had a significant positive impact on the self-rated health of older adults but not younger and middle-aged people. The self-rated health of urban residents was more positively impacted by Internet use than that of rural residents.

#### 3.4.4. Mediating Effect Test

To validate mediation pathways, we adopted the Wen and Ye mediation framework [32]. Regression analyses (Models 3–4) revealed significant associations: Internet use positively predicted physical exercise frequency (β = 0.269, *p* < 0.001), which in turn correlated with enhanced self-rated health (β = 0.031, *p* < 0.001). Given the ordered probit model specification, the Karlson-Holm-Breen (KHB) method [33] was implemented to decompose total effects into direct and indirect components—a critical advantage for nonlinear models where traditional linear mediation approaches face scaling limitations. Results from Table 7 indicate a total Internet use effect of 0.092 (*p* < 0.01) on SRH. The indirect contribution via physical exercise constituted 9% of this effect (direct: 0.084, *p* < 0.05; indirect: 0.008, *p* < 0.08), confirming partial mediation by leisure-time physical activity.

## 4. Discussion

### 4.1. Summary of Findings

This study employed survey data from the 2021 CGSS and an ordered probit model to systematically examine the mechanisms linking Internet use to self-rated health in adults. The analysis included mediation testing, subgroup heterogeneity exploration, and robustness checks. The key findings revealed were as follows: (1) a statistically significant positive association between Internet use and SRH; (2) a partial mediation of this relationship through physical exercise; (3) stratified effects, with urban residents and older adults demonstrating stronger SRH improvements compared to rural populations and younger cohorts.

Regarding the positive impact of the Internet on self-rated health, there are two mechanisms. One is that the interactive and personalized nature of the Internet offers individuals health benefits, and improves their quality of life and mental health [34]. Hartanto et al. [35] revealed that computer use longitudinally predicted better self-reported physical and mental health and reduced functional disabilities. The Internet broadens individuals’ horizons and knowledge, enabling them to access medical information and health consultations conveniently and to receive high-quality healthcare services. It allows individuals to acquire a wide range of entertainment and knowledge, expanding their perspectives and helping them explore and embrace a more fulfilling life [36,37,38]. The other is that when combined with offline elements like exercise and social contacts, Internet use had a positive effect on self-rated health [39,40]. The development of the Internet has enhanced communication channels with family and friends, enabling individuals to gain more emotional support and deepen their self-rated health [16].

As for the mediating role of exercise, prior research has yielded that the Internet increases people’s willingness to exercise and the frequency of their participation [24]. The empirical studies consistently demonstrate associations between digital engagement and physical activity patterns. For example, Wang et al. (2021), analyzing 2017 CGSS data, identified significantly higher moderate-to-vigorous physical activity levels among older adults with regular Internet use [41]. Parallel findings emerged from Kearns et al. (2019) in UK community-based surveys, reinforcing the observed relationship between technology adoption and exercise behaviors [42]. Physical exercise, aimed at improving fitness, regulating mental health, and enriching life, significantly impacts self-rated health [23]. Physical exercise mediates only 8.7% of the total effect. The small effect size likely reflects the multifactorial nature of SRH, where Internet use influences health through additional channels such as mental health support and healthcare access. Future research should explore complementary mediators, such as social capital and health literacy.

Elderly populations, characterized by restricted access to diversified information channels and diminished social connectivity, derive substantial benefits from online informational ecosystems that enhance societal engagement and expand interpersonal networks [43,44]. Digital platforms serve as critical facilitators of emotional fulfillment for this demographic, addressing their heightened dependence on sustained communication with familial and social circles [45]. Empirical evidence indicates that robust emotional support systems mediated through these technologies effectively mitigate psychological distress while enhancing well-being. The proliferation of digital health resources has transformed online platforms into essential tools for health literacy enhancement and preventive health management among aging populations [46]. Notably, elderly users leverage private messaging functionalities to selectively exchange health-related content, demonstrating active participation in knowledge dissemination practices [47]. The rapid spread of Internet knowledge leads to greater marginal health benefits for older adults compared to younger and middle-aged individuals.

China’s predominantly agrarian societal structure, characterized by a substantial rural population engaged in agricultural production, coexists with persistent developmental disparities in regional infrastructure. Systemic inequities in digital infrastructure investment between urban and rural zones have been observed, potentially constraining technological diffusion pathways and perpetuating connectivity deficits in peri-urban territories [48]. Rural residents generally have lower education levels and may not utilize Internet resources as efficiently as urban residents to obtain health information and adopt corresponding health behaviors. In contrast, urban residents are likely to use the Internet more frequently to search for health information, leading to a more comprehensive understanding of their health. Moreover, urban residents have better SRH, which may stem from superior healthcare access and digital literacy.

### 4.2. Policy Implications

Building on analytical insights and Grossman’s health capital theory—which posits sustained investment in health-promoting resources as critical to maintaining or decelerating the depreciation of health capital stocks [49]—this study advocates for Internet-enabled strategies to enhance physical activity participation among adults, particularly targeting rural residents and older populations with limited digital access. First, leverage digital platforms for disseminating fitness guidelines and fostering proactive health consciousness. The theory of network empowerment argues that online engagement enhances individuals’ self-efficacy and agency in health behavior regulation [50]. Access to digital health literacy illustrates this mechanism, whereby people optimize their perceived ability to access web-based guidance on physical activity, thus elevating both exercise initiation frequency and adherence duration. Second, expand Internet infrastructure to mitigate urban-rural and intergenerational connectivity gaps. Current disparities are stark: as of December 2023, rural users (326 million) comprised merely 29.8% of China’s Internet population, while individuals aged ≥ 60 years represented only 15.6% of total users, underscoring the urgency of inclusive digital expansion. In alignment with China’s strategic implementation of “Internet of Things” frameworks and intelligent healthcare systems [51,52], targeted enhancement of digital connectivity infrastructure may amplify health equality by bridging critical accessibility gaps, particularly in marginalized regions with limited technological penetration. This paradigm demonstrates particular relevance for underserved populations, where differentials in digital resource allocation disproportionately constrain healthcare access and exacerbate health disparities. Third, digitally mediated health interventions demonstrate significant potential for aging populations. For example, one study showed that text messaging was effective in promoting smoking cessation among Chinese adults [53]. Such a model is particularly promising in the context of China’s accelerating demographic transition, which is currently characterized by an extended longevity index and declining fertility. A scalable digital health initiative could address age-specific morbidity patterns while aligning with national public health priorities.

### 4.3. Limitations

This study acknowledges several methodological constraints. First, the cross-sectional design limits causal inference given the dynamic nature of health status evolution over time, necessitating future validation through longitudinal tracking. Second, the analysis did not differentiate between types of mobile Internet applications or usage duration, nor did it incorporate objective health metrics such as clinical biomarkers—gaps to be addressed in subsequent research. Third, measurement limitations arose from the generalized operationalization of Internet engagement variables, potentially introducing classification inaccuracies. Additionally, the marginal explanatory power of physical exercise as a mediator suggests limited practical significance in the observed Internet use–exercise-SRH relationship.

## 5. Conclusions

According to our research, Chinese peoples’ self-rated health is positively correlated with Internet use, which is mediated by their increased frequency of physical activity. This is consistent with China’s efforts to promote health governance in recent years through its Internet Plus Exercise strategy and provides public health policy insights for other countries around the globe. Future studies can further focus on Internet quality and information content and use longitudinal data to examine the causal relationship between the three.

## Figures and Tables

**Table 1:** Descriptive statistics.

Variables (Percent)	Mean	SD
**Dependent variable**		
**SRH** Poor = 1(5.26%), Fairly poor = 2(13.18%), Average = 3(28.07%), Fairly good = 4(35.32%), Good = 5(18.17%)	3.480	1.090
**Independent variable** **INT** Yes = 1(71.84%), No = 0(28.16%)	0.720	0.450
**Leisure** Never = 1(31.03%), Rarely = 2(2.61%), Sometimes = 3(3.07%), Often = 4(7.82%), Very often = 5(55.46%)	3.540	1.810
**Mediating Variables** **Physical exercise** Never = 0(35.18%), Not regularly = 1(24.95%), Regularly = 2(15.79%), Daily = 3(24.08%)	1.290	1.180
**Control variables**		
**Age** 18–44 = 0(33.98%), 45–59 = 1(30.26%), 60 and above = 2(35.77%)	1.020	0.830
**Gender** Female = 0(54.55%), Male = 1(45.45%)	0.450	0.500
**Hukou** Rural = 1(60.14%), Urban = 0(39.86%)	0.600	0.490
**Marital status** Married = 1(72.07%), Otherwise = 0(27.93%)	0.720	0.450
**Work** Never worked = 0(7.31%), Unemployment = 1(41.41%), Agricultural work = 2(15.85%), Non-agricultural work = 3(35.43%)	1.790	1.010
**Health** Poor = 1(4.99%), Fairly poor = 2(11.45%), Average = 3(15.21%), Fairly good = 4(22.21%), Good = 5(46.15%)	3.930	1.230
**Education** Primary school and below = 0(33%), Junior/Senior high school = 1(46.86%), College and above = 2(20.14%)	0.870	0.720

**Table 2:** Correlation analysis of variables (Spearman).

	SRH	INT	Leisure	Exercise
**SRH**	1			
**INT**	0.272 ***	1		
**Leisure**	0.298 ***	0.802 ***	1	
**Exercise**	0.137 ***	0.184 ***	0.224 ***	1

*** *p* < 0.001.

**Table 3:** Multicollinearity test results.

Variables	VIF
INT	1.61
Exercise	1.12
Age	1.93
Education	1.77
Hukou	1.25
Work	1.23
Health	1.18
Marital	1.08
Gender	1.05

**Table 4:** Baseline regression results.

Variable	Model1	Model2	Model3	Model4
	SRH	SRH	Exercise	SRH
**INT**	0.656 *** (0.0280)	0.119 *** (0.0359)	0.269 *** (0.0381)	0.0319 ** (0.0112)
**Exercise**				0.111 ** (0.0359)
**Age (ref:18–44)**				
45–59		−0.325 *** (0.0362)	0.181 *** (0.0366)	−0.332 *** (0.0363)
60 and above		−0.366 *** (0.0443)	0.295 *** (0.0457)	−0.376 *** (0.0444)
Rural ** (ref: Urban)**		0.0659 * (0.0304)	−0.250 *** (0.0307)	0.0745 * (0.0305)
Male ** (ref: Female)**		0.0351 (0.0262)	0.0709 ** (0.0266)	0.0409 (0.0260)
Married ** (ref: Otherwise)**		−0.0437 (0.0301)	−0.0630 (0.0390)	−0.0439 (0.0301)
**Work (ref: Never worked)**				
Unemployment		−0.224 *** (0.0558)	−0.110 (0.0748)	−0.221 *** (0.0559)
Agricultural work		−0.165 ** (0.0626)	−0.259 ** (0.0816)	−0.155 * (0.0627)
Non-agricultural work		−0.0790 (0.0541)	−0.107 (0.0751)	−0.0715 (0.0542)
**Health (ref: Poor)**				
Fairly poor		0.675 *** (0.0694)	0.219 ** (0.0741)	0.670 *** (0.0694)
Average		1.511 *** (0.0694)	0.341 *** (0.0715)	1.505 *** (0.0694)
Fairly good		2.129 *** (0.0693)	0.430 *** (0.0698)	2.120 *** (0.0694)
Good		2.538 *** (0.0677)	0.505 *** (0.0676)	2.526 *** (0.0678)
**Education (ref: Primary and below)**				
Junior/Senior high school		0.0828 * (0.0327)	0.359 *** (0.0342)	0.0724 * (0.0329)
College and above		0.129 ** (0.0480)	0.568 *** (0.0488)	0.112 * (0.0484)
**Provinces**	Yes	Yes	Yes	Yes
**Observations**	7582	7582	7582	7582

* *p* < 0.05, ** *p* < 0.01, *** *p* < 0.001.

**Table 5:** Robustness test results of Internet use on self-rated health.

Variable	Model5	Model6	Model7	Model8
	SRH	SRH	SRH	SRH
**INT**	1.152 *** (0.0495)	0.215 *** (0.0629)		
**Leisure**			0.177 *** (0.00701)	0.0351 *** (0.00924)
**Age (ref:18–44)**				
45–59		−0.561 *** (0.0623)		−0.314 *** (0.0365)
60 and above		−0.623 *** (0.0770)		−0.352 *** (0.0449)
Rural ** (ref: Urban)**		0.106 * (0.0523)		0.0663 * (0.0304)
Male ** (ref: Female)**		0.0647 (0.0448)		0.0419 (0.0260)
Married ** (ref: Otherwise)**		−0.102 * (0.0521)		−0.0101 * (0.0532)
**Work (ref: Never worked)**				
Unemployment		−0.387 *** (0.0962)		−0.226 *** (0.0559)
Agricultural work		−0.281 ** (0.108)		−0.164 ** (0.0626)
Non-agricultural work		−0.145 (0.0924)		−0.0826 (0.0541)
**Health (ref: Poor)**				
Fairly poor		1.324 *** (0.124)		0.680 *** (0.0694)
Average		2.886 *** (0.128)		1.514 *** (0.0693)
Fairly good		3.996 *** (0.130)		2.134 *** (0.0692)
Good		4.694 *** (0.128)		2.540 *** (0.0676)
**Education (ref: Primary and below)**				
Junior/Senior high school		0.147 ** (0.0572)		0.0782 * (0.0327)
College and above		0.195 * (0.0829)		0.119 * (0.0483)
Provinces	Yes	Yes	Yes	Yes
Observations	7582	7582	7582	7582

* *p* < 0.05, ** *p* < 0.01, *** *p* < 0.001.

**Table 6:** Heterogeneity test for different ages and different household registration.

Variables	Age			Hukou	
	18–45	46–59	Age ≥ 60	Urben	Rural
INT	0.221 (0.187)	0.0792 (0.0609)	0.105 * (0.0475)	0.173 ** (0.0631)	0.0834 (0.0444)
Provinces	Yes	Yes	Yes	Yes	Yes
Control variables	Yes	Yes	Yes	Yes	Yes
Observations	2576	2576	2294	3022	4560

* *p* < 0.05, ** *p* < 0.01.

**Table 7:** The results of the KHB test.

	Coeff	SE	P	Amount of Effect (%)
Total Effect	0.092 **	0.029	0.008	
Direct Effect	0.084 *	0.035	0.015	91.3%
Indirect Effect	0.008 **	0.031	0.008	8.7%

* *p* < 0.05, ** *p* < 0.01.

## Data Availability

Data are contained within the article.

## References

[1] The 53rd Statistical Report on China’s Internet Development. https://www.cnnic.com.cn/IDR/ReportDownloads/202405/P020240509518443205347.pdf.

[2] He D., Gu Y., Shi Y., Wang M., Lou Z., Jin C. (2020). COVID-19 in China: The role and activities of Internet-based healthcare platforms. Glob. Health Med..

[3] Yang F., Shu H., Zhang X. (2021). Understanding “internet plus healthcare” in china: Policy text analysis. J. Med. Internet Res..

[4] Zhou D.S., Zhan Q.Q., Wen X. (2023). How does digital life influence the health service use among rural residents? Evidence from China. Technol. Health Care.

[5] Ghahramani F., Wang J. (2020). Impact of Smartphones on Quality of Life: A Health Information Behavior Perspective. Inf. Syst. Front..

[6] Wang J.L., Wu H.T., Liu Y., Wang W.L. (2024). Health welfare in the digital era: Exploring the impact of digital trade on residents’ health. Econ. Hum. Biol..

[7] Wen W., Zhang Y., Shi W., Li J. (2023). Association Between Internet Use and Physical Health, Mental Health, and Subjective Health in Middle-aged and Older Adults: Nationally Representative Cross-sectional Survey in China. J. Med. Internet Res..

[8] Li L., Ding H. (2022). The Relationship between Internet Use and Population Health: A Cross-Sectional Survey in China. Int. J. Environ. Res. Public Health.

[9] Frison E., Eggermont S. (2015). Exploring the Relationships Between Different Types of Facebook Use, Perceived Online Social Support, and Adolescents’ Depressed Mood. Soc. Sci. Comput. Rev..

[10] Lee H.Y., Kim J., Sharratt M. (2018). Technology use and its association with health and depressive symptoms in older cancer survivors. Qual. Life Res..

[11] Liu Y., Ni X., Niu G. (2020). The influence of active social networking services use and social capital on flourishing in Chinese adolescents. Child. Youth Serv. Rev..

[12] Bevilacqua R., Strano S., Di Rosa M., Giammarchi C., Cerna K.K., Mueller C., Maranesi E. (2021). eHealth Literacy: From Theory to Clinical Application for Digital Health Improvement. Results from the ACCESS Training Experience. Int. J. Environ. Res. Public Health.

[13] Liu N., He Y., Li Z. (2022). The Relationship between Internet Use and Self-Rated Health among Older Adults in China: The Mediating Role of Social Support. Int. J. Environ. Res. Public Health.

[14] Rosenthal S.R., Buka S.L., Marshall B.D.L., Carey K.B., Clark M.A. (2016). Negative Experiences on Facebook and Depressive Symptoms Among Young Adults. J. Adolesc. Health.

[15] Lee S.Y. (2014). How do people compare themselves with others on social network sites?: The case of Facebook. Comput. Hum. Behav..

[16] Zhang L., Li S., Ren Y. (2024). Does internet use benefit the mental health of older adults? Empirical evidence from the China health and retirement longitudinal study. Heliyon.

[17] Lewandowski J., Rosenberg B.D., Jordan Parks M., Siegel J.T. (2011). The effect of informal social support: Face-to-face versus computer-mediated communication. Comput. Hum. Behav..

[18] Tang D., Jin Y., Zhang K., Wang D. (2022). Internet Use, Social Networks, and Loneliness Among the Older Population in China. Front. Psychol..

[19] Bull F.C., Al-Ansari S.S., Biddle S., Borodulin K., Buman M.P., Cardon G., Carty C., Chaput J.P., Chastin S., Chou R.G. (2020). World Health Organization 2020 guidelines on physical activity and sedentary behaviour. Br. J. Sports Med..

[20] Zhang S., Zhang Y.J. (2021). The Relationship Between Internet Use and Mental Health Among Older Adults in China: The Mediating Role of Physical Exercise. Risk Manag. Healthc. Policy.

[21] Hansen A.W., Beyer N., Flensborg-Madsen T., Gronbæk M., Helge J.W. (2013). Muscle strength and physical activity are associated with self-rated health in an adult Danish population. Prev. Med..

[22] Engberg E., Liira H., Kukkonen-Harjula K., From S., Kautiainen H., Pitkälä K., Tikkanen H. (2015). Associations of physical activity with self-rated health and well-being in middle-aged Finnish men. Scand. J. Public Health.

[23] Ibsen B., Elmose-Osterlund K., Hoyer-Kruse J. (2024). Associations of types of physical activity with self-rated physical and mental health in Denmark. Prev. Med. Rep..

[24] Guo B., Zhang X.D., Zhang R., Chen G. (2022). The Association between Internet Use and Physical Exercise among Middle-Aged and Older Adults-Evidence from China. Int. J. Environ. Res. Public Health.

[25] Li L., Ding H. (2022). Internet Use, Leisure Time and Physical Exercise of Rural Residents—Empirical Analysis Based on 2018 CFPS Data. Lanzhou Acad. J..

[26] Chen N., Shen Y., Liang H., Guo R. (2021). Housing and Adult Health: Evidence from Chinese General Social Survey (CGSS). Int. J. Environ. Res. Public Health.

[27] Ding H., Zhang C., Xiong W. (2022). Associations between Mobile Internet Use and Self-Rated and Mental Health of the Chinese Population: Evidence from China Family Panel Studies 2020. Behav. Sci..

[28] Li Z., Wang Y., Li X., Luo Y. (2023). Research on the Correlation between Digital Health Literacy and Physical Health Status in Middle-Aged and Elderly Adults: Based on the Mediating Effect of Physical Exercise Behavior. China Sport Sci. Technol..

[29] Lu J., Wang B. (2020). The Mechanism of the Impact of Internet Use on Residents’ Self-rated Health: Based on the 2016 China Family Panel Studies. J. Sun Yat-Sen Univ. (Soc. Sci. Ed.).

[30] Zhang X., Wang D., Li F. (2023). Physical Exercise, Social Capital, Hope, and Subjective Well-Being in China: A Parallel Mediation Analysis. Int. J. Environ. Res. Public Health.

[31] Sui M., Ding H., Xu B., Zhou M. (2022). The Impact of Internet Use on the Happiness of Chinese Civil Servants: A Mediation Analysis Based on Self-Rated Health. Int. J. Environ. Res. Public Health.

[32] Wen Z., Ye B. (2014). Analyses of Mediating Effects: The Development of Methods and Models. Adv. Psychol. Sci..

[33] Kohler U., Karlson K.B., Holm A. (2011). Comparing coefficients of nested nonlinear probability models. Stata J..

[34] Ahn J.-H., Lim K.-C., Lee Y.-J., Kim K.-S. (2011). Effects of computer/internet game play on depression and life satisfaction among the elderly: Mediating effects of perceived self-control. J. Korea Contents Assoc..

[35] Hartanto A., Yong J.C., Toh W.X., Lee S.T., Tng G.Y., Tov W. (2020). Cognitive, social, emotional, and subjective health benefits of computer use in adults: A 9-year longitudinal study from the Midlife in the United States (MIDUS). Comput. Hum. Behav..

[36] Luo X., Pu H., Wang S., Zhong D., Liu F., Li Z. (2024). Influence of Internet use on Chinese residents’ health: The mediating role of health knowledge. Technol. Soc..

[37] Nevado-Peña D., López-Ruiz V.-R., Alfaro-Navarro J.-L. (2019). Improving quality of life perception with ICT use and technological capacity in Europe. Technol. Forecast. Soc. Change.

[38] Peng Y.-I., Chan Y.-S. (2020). Do internet users lead a healthier lifestyle?. J. Appl. Gerontol..

[39] Kobayashi L.C., Wardle J., von Wagner C. (2015). Internet use, social engagement and health literacy decline during ageing in a longitudinal cohort of older English adults. J. Epidemiol. Community Health.

[40] Chen H., Zhang T.P.M., Li Y.H., Zhao W.F., Xu W. (2024). Relationship and mechanisms between internet use and physical exercise among middle- and younger-aged groups. PLoS ONE.

[41] Wang S., Guo K., Lu W. (2021). Will internet use promote physical exercise for the elderly in China? An empirical analysis based on CGSS. J. Sports Res..

[42] Kearns A., Whitley E. (2019). Associations of internet access with social integration, wellbeing and physical activity among adults in deprived communities: Evidence from a household survey. BMC Public Health.

[43] Chen W.-C., Yang L., Wang X.-Y. (2022). Internet Use, Cultural Engagement, and Multi-Dimensional Health of Older Adults: A Cross-Sectional Study in China. Front. Public Health.

[44] Li S.J., Cui G.H., Yin Y.T., Xu H.L. (2023). Associations between health literacy, digital skill, and eHealth literacy among older Chinese adults: A cross-sectional study. Digit. Health.

[45] Xie B. (2008). Multimodal computer-mediated communication and social support among older Chinese internet users. J. Comput. Mediat. Commun..

[46] Yong W., Zhanhong Z., Xingyu S., Jianfang Z., Xiaoming S., Xiaomei R. (2014). Health status, health needs and provision of health services among the middle-aged and elderly people. Popul. Res..

[47] Saleh J., Robinson B.S., Kugler N.W., Illingworth K.D., Patel P., Saleh K.J. (2012). Effect of social media in health care and orthopedic surgery. Orthopedics.

[48] Hong Y.A., Zhou Z., Fang Y., Shi L. (2017). The digital divide and health disparities in China: Evidence from a national survey and policy implications. J. Med. Internet Res..

[49] Grossman M. (2017). The Demand for Health: A Theoretical and Empirical Investigation.

[50] Hanson J. (2014). Empowerment and online social networking. The Handbook of Media and Mass Communication Theory.

[51] Zheng X. (2015). Rodríguez-Monroy C: The development of intelligent healthcare in China. Telemed. e-Health.

[52] Hvistendahl M. (2012). China Pushes the ‘Internet of Things’.

[53] Liao Y., Wu Q., Tang J., Zhang F., Wang X., Qi C., He H., Long J., Kelly B.C., Cohen J. (2016). The efficacy of mobile phone-based text message interventions (‘Happy Quit’) for smoking cessation in China. BMC Public Health.

